# Screening of high-risk deleterious missense variations in the *CYP1B1* gene implicated in the pathogenesis of primary congenital glaucoma: A comprehensive *in silico* approach

**DOI:** 10.7717/peerj.14132

**Published:** 2022-11-30

**Authors:** Muhammad Shahid, Ahmad Azfaralariff, Muhammad Tufail, Nazeer Hussain Khan, Ahmed Abdulkareem Najm, Sabika Firasat, Muhammad Zubair, Shazrul Fazry, Douglas Law

**Affiliations:** 1 Department of Biological Sciences and Biotechnology, Faculty of Science and Technology, Universiti Kebangsaan Malaysia, Bangi, Selangor, Malaysia; 2 Department of Food Sciences, Faculty of Science and Technology, Universiti Kebangsaan Malaysia, Bangi, Selangor, Malaysia; 3 Department of Zoology, Faculty of Biological Sciences, Quaid-i-Azam University, Islamabad, Pakistan; 4 School of Life Sciences, Henan University, Kaifeng, Henan, China; 5 Department of Wildlife and Ecology, University of Veterinary and Animal Sciences, Pattoki Campus, Pattoki, Punjab, Pakistan; 6 Tasik Chini Research Center, Faculty of Science and Technology, Universiti Kebangsaan Malaysia, Bangi, Selangor, Malaysia; 7 Faculty of Health and Life Sciences, Inti International University, Persiaran Perdana BBN Putra Nilai, Nilai, Negeri Sembilan, Malaysia

**Keywords:** Primary congenital glaucoma, CYP1B1, Missense variations, *In silico*, Modeling, Molecular dynamic simulation

## Abstract

**Background:**

Primary congenital glaucoma (PCG) is the most common subtype of glaucoma caused by defects in the cytochrome P450 1B1 (CYP1B1) gene. It is developing among infants in more than 80% of cases who exhibit impairments in the anterior chamber angle and the trabecular meshwork. Thus, a comprehensive *in silico* approach was performed to evaluate the effect of high-risk deleterious missense variations in the *CYP1B1* gene.

**Material and methods:**

All the information for *CYP1B1* missense variants was retrieved from the dbSNP database. Seven different tools, namely: SIFT, PolyPhen-2, PROVEAN, SNAP2, PANTHER, PhD-SNP, and Predict-SNP, were used for functional annotation, and two packages, which were I-Mutant 2.0 and MUpro, were used to predict the effect of the variants on protein stability. A phylogenetic conservation analysis using deleterious variants was performed by the ConSurf server. The 3D structures of the wild-type and mutants were generated using the I-TASSER tool, and a 50 ns molecular dynamic simulation (MDS) was executed using the GROMACS webserver to determine the stability of mutants compared to the native protein. Co-expression, protein-protein interaction (PPI), gene ontology (GO), and pathway analyses were additionally performed for the CYP1B1 in-depth study.

**Results:**

All the retrieved data from the dbSNP database was subjected to functional, structural, and phylogenetic analysis. From the conducted analyses, a total of 19 high-risk variants (P52L, G61E, G90R, P118L, E173K, D291G, Y349D, G365W, G365R, R368H, R368C, D374N, N423Y, D430E, P442A, R444Q, F445L, R469W, and C470Y) were screened out that were considered to be deleterious to the CYP1B1 gene. The phylogenetic analysis revealed that the majority of the variants occurred in highly conserved regions. The MD simulation analysis exhibited that all mutants’ average root mean square deviation (RMSD) values were higher compared to the wild-type protein, which could potentially cause CYP1B1 protein dysfunction, leading to the severity of the disease. Moreover, it has been discovered that CYP1A1, VCAN, HSD17B1, HSD17B2, and AKR1C3 are highly co-expressed and interact with CYP1B1. Besides, the CYP1B1 protein is primarily involved in the metabolism of xenobiotics, chemical carcinogenesis, the retinal metabolic process, and steroid hormone biosynthesis pathways, demonstrating its multifaceted and important roles.

**Discussion:**

This is the first comprehensive study that adds essential information to the ongoing efforts to understand the crucial role of genetic signatures in the development of PCG and will be useful for more targeted gene-disease association studies.

## Introduction

Glaucoma is a heterogeneous group of complex diseases that gradually lead to permanent loss of vision. It is estimated that 79.6 million people worldwide were affected in 2020, and the number is predicted to rise to 111.8 million by 2040 (Quigley & Broman, 2006; Tham et al., 2014). The modern classification categorizes glaucoma into three groups, which are: primary congenital glaucoma (PCG); primary open-angle glaucoma (POAG); and primary angle closure glaucoma (PACG). The most common subtype of glaucoma is PCG, and it has been identified as developing among infants in more than 80% of cases. This disorder develops due to impairments in the anterior chamber angle and the trabecular meshwork. Clinically, the affected patients manifest corneal edema, photophobia, epiphora (watery eye), and buphthalmolos (enlargement of the globe), which result from elevated intraocular pressure (IOP) (Vasiliou & Gonzalez, 2008). The increased of IOP in untreated individuals with PCG is a major risk factor that leads to rapid axonal loss and permanent loss of vision (Vasiliou & Gonzalez, 2008). The incidence rate of PCG are varies depending on ethnic and geographical groups, where 1:1250 has been reported in the Slovak Roman population; 1:10000 in Western countries and 1:2500 in the Middle East (Badawi et al., 2019; Gencik, Gencikova & Ferak, 1982).

PCG is an autosomal recessive disorder that has a variable penetrance rate. It more frequently affects males at around 65% compared to females at 35%, demonstrating the sporadic transmission (in 90% of cases), but pseudodominant transmission has also been shown in some families (Bejjani et al., 2000). Through genetic linkage analysis, four loci of PCG have been discovered, which were GLC3A, GLC3B, GLC3C, and GLC3D, on chromosomes 2p22-p21, 1p36.2-p36.1, 14q24.3, and 14q24, respectively (Akarsu et al., 1996; Firasat et al., 2008; Sarfarazi et al., 1995; Stoilov, MJIo & v, 2002). In addition, two genes, *CYP1B1* (coding for cytochrome P450, family 1, subfamily B, polypeptide 1) and, *LTBP2* (coding for latent transforming growth factor *β*-binding protein 2) were recently discovered. *CYP1B1* was found on GLC3A loci, while *LTBP2* was found on GLC3D loci (Ali et al., 2009; Stoilov, Akarsu & Sarfarazi, 1997). Molecular screening revealed that *CYP1B1* is a more mutated gene compared to *LTBP2*, and hundreds of variations have been reported in this gene that are associated with PCG phenotypes (Rashid et al., 2019).

*CYP1B1* belongs to the CYP450 superfamily and genomic DNA length of 8.5 kb. This gene is expressed in the eye, kidney, breast, prostate, and brain tissues (Muskhelishvili et al., 2001). It is also known as the first gene predominantly associated with primary developmental defects (Muskhelishvili et al., 2001). Functionally, it is a member of the aryl hydrocarbon receptor (AHR) battery that is incorporated into both endogenous and exogenous substrate metabolism (Tang et al., 1996). It is exclusively involved in the metabolism of carcinogens, steroids, arachidonate, retinol, retinal, and melatonin that may trigger signal transduction pathways to control the growth and differentiation of tissues. Through these corresponding mechanisms, *CYP1B1* probably regulates the early ocular differentiation processes. Recent studies found that *CYP1B1* is also expressed constitutively in vascular cells, which play an important role during postnatal retinal vascular development (Tang et al., 2009). Apart from that, several studies conducted on humans and mice have elucidated that *CYP1B1* plays a pivotal role in the proper development of trabecular meshwork and other ocular tissues. Besides PCG, *CYP1B1* has also been linked with other diseases, including various types of cancer and cardiovascular diseases (Falero-Perez et al., 2018).

In the development and progression of a disease, several environmental and genetic factors are involved. Previous research found that single nucleotide polymorphisms (SNPs) are the most common genetic determinants, occurring in one out of every 3000 base pairs in the human genome and serving as potential biomarkers for a variety of life-threatening diseases (Earl & Greenhalf, 2009). Among all SNPs, the presence of non-synonymous SNPs (nsSNPs), also called missense variations, is considered to have profound phenotypic effects upon a variation in protein amino acid sequence. It has been seen that these missense variations have substantial potential to alter the structure, stability, biological functions, and interactions of the protein (Bhattacharya & Ray, 2021). Therefore, numerous *in silico* studies have been carried out previously to explore the deleterious missense variation in many genes associated with various diseases’ pathogenesis (Agrahari et al., 2019; Bhattacharya & Ray, 2021; Shahid et al., 2022; Sunkar & Neeharika, 2020). The identification of deleterious missense variations in the *CYP1B1* gene is absolutely essential for more detailed comprehension to identify novel biomarkers in the prognosis and management of PCG. Therefore, this study aimed to investigate the structural and functional effects of the high-risk deleterious missense variants of *CYP1B1* using various computational tools.

## Material and Methods

### Data retrieval

The list of missense variations in the *CYP1B1* gene of *Homo sapiens* was retrieved from the dbSNP database of the National Center for Biotechnology Information (NCBI) (http://www.ncbi.nlm.nih.gov/projects/SNP/). The encoded protein sequence with reference ID: NP_000095.2 was also downloaded from the same database.

### Functional annotation of variants

The experiment is carried out computationally and is called the “*in silico* method.” These methods use databases, network analysis, and machine learning approaches that are faster, more critical, and more cost-effective than *in vitro* and *in vivo* studies (Azfaralariff et al., 2022). Therefore, in the present study, to identify the high-risk deleterious variations, the functional annotation of missense variations in the *CYP1B1* gene was carried out by employing multiple bioinformatics tools. Seven different tools, which were SIFT, PolyPhen-2, PROVEAN, SNAP2, PANTHER, PhD-SNP, and Predict-SNP, were used in this screening assay. The first screening tool employed in this assay to investigate the deleterious variants from the data retrieved from the dbSNP NCBI database was called SIFT (sorting intolerant from tolerant) (https://sift.bii.a-star.edu.sg/). It predicts the functional importance of the altered amino acid substitution using evolutionary conservation *via* sequence homology alignment and the physiochemical characteristics of a protein. The SIFT algorithm predicts substitutions with a probability score ≥ 0.05 as tolerated substitutions, while those with a probability score <0.05 are considered deleterious (Ng & Henikoff, 2003; Shahid et al., 2020). Polyphen 2.0 (polymorphism phenotyping v2) (http://genetics.bwh.harvard.edu/pph2/) was utilized, acknowledging its structural and functional impact prediction of a single amino acid alteration in a protein. This tool evaluates an amino acid substitution using comparative homology protein models and generates a position specific independent count (PSIC) score. It annotates a substitution as probably damaging, possibly damaging, or benign if the probability score is >0.85, >0.15, and <0.15, respectively (Adzhubei et al., 2010). Further, the PROVEAN (Protein Variation Effect Analyzer) (http://provean.jcvi.org/index.php) server was accessed in this functional annotation assay. This server predicts the functional impact of an amino acid alteration in a protein and categorizes them as neutral or deleterious, if the generated score is greater than −2.5 or less than −2.5, respectively (Choi et al., 2012). The next screening was performed by the PANTHER (Protein analysis through evolutionary relationship-position-specific evolutionary preservation) (http://www.pantherdb.org/) server. This tool calculates the score of an amino acid variant based on evolutionary preservation (time in millions of years) using the HMM algorithm. It annotates an amino acid alteration as benign: time <200 my, possibly damaging: 450 my to 200 my, or probably damaging: 450 my, respectively (Tang & Thomas, 2016). SNAP2 (Screening of non-acceptable Polymorphism) (https://rostlab.org/services/snap2web/) tool was employed to determine the pathogenicity of an amino acid alteration in a protein. It is an ensemble of neural network models that predicts both the clinical and molecular effects of a missense variant in the form of strongly effective (score: +100) or strongly neutral (score: −100) (Bromberg & Rost, 2007). Moreover, the PhD-SNP (Predictor of human deleterious single nucleotide polymorphism) tool, accessed from https://snps.biofold.org/phd-snp/phd-snp.html, was also utilized in this functional assay. It is a Support Vector Machine (SVM) tool that predicts the pathogenicity of an amino acid substitution in a protein based on evolutionary information and a hybrid predictive model. This tool classifies the missense variants as neutral or pathogenic and provides a reliability index (RI) ranging from 0-10, with 10 being the highest reliability (Capriotti, Calabrese & Casadio, 2006). Additionally, the Predict-SNP (https://loschmidt.chemi.muni.cz/predictsnp/) tool was also approached to predict the disease related variations of the *CYP1B1* gene. It uses consensus-based methods to generate its output (Bendl et al., 2014). The allelic frequency of the variants was determined using the gnomAD (Genome Aggregation Database) version 2.1.1 (https://gnomad.broadinstitute.org/). The gnomAD is the most widely used database that harbors the largest collection of sequencing data for population variations (Gudmundsson et al., 2022).

### Effect of variations on CYP1B1 protein stability

To evaluate the stability of the protein upon any amino acid alteration, two machine learning tools, namely, I-Mutant 2.0 and MUpro, were utilized. I-Mutant 2.0 (https://folding.biofold.org/i-mutant/i-mutant2.0.html) predicts protein stability using an SVM-based algorithm and calculates the amino acid change in the form of Gibbs free energy (DDG) at a given pH and temperature. It categorizes the protein stability as increased or decreased and provides a reliability index (RI) ranging from 0 to 10, with 10 being the highest reliability (Capriotti, Fariselli & Casadio, 2005). The input conditions were set to pH 7 and temperature 37 °C respectively. Additionally, the MUpro server (http://mupro.proteomics.ics.uci.edu/) was approached to validate the I-Mutant 2.0 outcomes. This server ensembles both SVM and neural network algorithms to predict the effect of amino acid alteration in a protein and classifies the protein stability as increased or decreased if the confidence scores are <0 or >0, respectively (Cheng et al., 2006).

### Conservancy analysis of high-risk variations and secondary structure of CY1B1

The evolutionary conservation of the high risk deleterious amino acid variations was evaluated by utilizing the ConSurf server (https://consurf.tau.ac.il/). This server works on the empirical Bayesian method and multiple sequence alignment approach to estimate the phylogenetic conservation score, which ranges from 1–9. Residues of conservation scores of 7–9 were considered highly conserved, 4–6 as intermediate, and 1–3 were represented as variable and less conserved. In addition, this tool also predicted the characteristics of residues based on their positions in the protein’s structure: either buried (b), exposed (e), highly conserved and exposed (functional, f), or highly conserved and buried (s), which further embellished the structural and functional significance of amino acid residues in a protein (Ashkenazy et al., 2010; Shahid et al., 2022). For the secondary structure prediction of CYP1B1 protein, the SOPMA (Self-optimized Prediction Method with Alignment) bioinformatic tool was utilized by using its default query parameters (window width, 17; similarity threshold, 8; and number of states, 4). This server ensembles the self-optimized prediction method and a neural network method (PHD) to generate the secondary structure of a protein (Geourjon & Deleage, 1995).

### Structural examination of native and mutant types of CYP1B1

The three-dimensional (3D) structure of CYP1B1 protein was downloaded from the Protein Data Bank (PDB) (https://www.rcsb.org/). To generate the complete structure of the CYP1B1 protein, the I-TASSER (Iterative Threading Assembly Refinement) (https://zhanggroup.org/I-TASSER/) most advanced biological server was approached (Zhang, 2008). This is a tool that constructs the 3D structures of proteins through hierarchical approaches by first searching the templates from the PDB database and then constructing the models through iterative template fragment assembly simulation. Moreover, 19 mutant structures of CYP1B1 protein were also generated through the I-TASSER tool by incorporating the amino acid alteration of each high-risk deleterious variation into the wild-type FASTA protein sequence. The quality (degree of appropriateness) of all the native and mutant structures was verified through the ERRAT and VERIFY3D, which are in-built tools of the SAVES meta-server version 6.0 (https://saves.mbi.ucla.edu/). Subsequently, the resultant structures were further subjected to the TM-Align server (https://zhanglab.dcmb.med.umich.edu/TM-align/) which calculates the RMSD (Root Mean Square Deviation) to analyze the deviation of mutant structures from the wild-type (Zhang & Skolnick, 2005). Graphical inspection and superimposition of wild-type and mutant structures were performed by UCSF Chimera software version 1.14 (Pettersen et al., 2004).

### Molecular dynamics simulation (MDS) analysis

The molecular dynamic simulation (MDS) for the native and mutant CYP1B1 protein structures was carried out using GROMACS simulation software accessed from https://simlab.uams.edu (Bekker et al., 1993). For the MDS run, the wild-type and mutant protein structures were prepared using the GROMOS96 43a1 force field solvated by the Single Point Charge (SPC) water model and fixed in a periodic cubic solvated box. Other requisite parameters in molecular dynamic simulation, such as neutralization by adding the salt (NaCl) concentration (0.15 M), energy minimization for 5,000 steps of the prepared system using the steepest descent method, equilibration type consisting of NVT/NPT at 300 K and 1 bar of pressure using the leap-frog method. The final MD simulation step was processed for 50 ns to analyze the deviation of mutant structures from the wild-type protein structure.

### Investigation of CYP1B1-interacting candidates

The co-expression analysis of the *CYP1B1* gene was evaluated through the GeneMANIA server (https://genemania.org/). It is a publically available bioinformatics tool that harbors a large database for gene functional analysis, including co-expression, pathway interaction, physical interaction, co-localization, genetic interaction, and shared protein domains (Shahid et al., 2021; Warde-Farley et al., 2010). The submitted query was run by setting *Homo sapiens* as the species organism and the maximum number of resultant genes and attributes as 10. On top of that, the protein-protein interaction (PPI) of CYP1B1 was also determined by utilizing the STRING protein database (https://string-db.org/). The STRING database query terms were cut-off at 0.7 and the maximum additional interaction was 10 (Azfaralariff et al., 2022; Szklarczyk et al., 2021). Cytoscape software version 3.8.2 (https://cytoscape.org/) was employed to construct the highly co-expressed genes and PPI networks in the present study (Shannon et al., 2003).

### Gene ontology and pathway enrichment analysis of CYP1B1

The functional annotation of three gene ontology (GO) terms, which were molecular functions (MF), cellular components (CC), and biological processes (BP), and the KEGG (Kyoto Encyclopedia of Genes and Genomes) pathway of CYP1B1 were analysed with Enrichr database (https://maayanlab.cloud/Enrichr/), a publically available online tool for gene expression profiling, transcriptional factor analysis, PPI networks, pathway analysis, toxicogenomics and pharmacogenomics studies (Chen et al., 2013). The *P*-value <0.05 was set as a cut-off criterion for the submitted query. Cytoscape software was utilized to construct all the networks.

## Results

### Data retrieval and selection

The dbSNP is a unique database that holds all the necessary information, including the variation data of many of the human genes. This database with *CYP1B1* reported a total of 4788 SNPs, of which 610 were missense, 276 were synonymous, 1359 were in the intronic region, and the rest belonged to other categories (Accessed: December 21, 2021). In this study, the missense variations were selected in order to investigate their damaging effects and how they influence the phenotypic characteristics of the respective encoded protein. These missense variations are responsible for various complex diseases by affecting the protein’s functional activities and structural stability or folding (Khalid & Almaghrabi, 2020; Shahid et al., 2022). A list of all 610 missense variants is presented in Table S1.

### Functional annotation of missense variants

The missense variations of a gene can produce amino acid allelic variants that may influence the functions of that particular gene product by disrupting the structural conformation. Hence, missense variants of the *CYP1B1* gene were chosen for further investigation. Variations of 610 *CYP1B1* missense retrieved from the dbSNP NCBI database were first subjected to the SIFT server, which predicted 92 variants to be either deleterious or tolerated (Table 1; Table S1 ). These 92 variants were further evaluated by six other computational tools, namely PolyPhen-2, PhD-SNP, SNAP2, PANTHER, PROVEAN, and Predict-SNP, which used different algorithms to scrutinize a variant. The predictions and categories of these tools are provided in Table 1. From conducted analysis, a total of 19 missense variants (P52L, G61E, G90R, P118L, E173K, D291G, Y349D, G365W, G365R, R368H, R368C, D374N, N423Y, D430E, P442A, R444Q, F445L, R469W, and C470Y) were found to be common in all the functional analysis tools by manual concordance, and all were deleterious, probable damaging, and effect variants. Therefore, these 19 variants were considered the high-risk deleterious variants of the *CYP1B1* gene and are promising candidates for further research. The detailed results of 19 high-risk variants are presented in Table 2 and their allele frequencies information are in Table 3.

**Table 1:** Prediction of functional consequences of the total number of variants of *CYP1B1* gene by different computational tools.

Tool	Prediction	Software outputs
SIFT	Tolerated	38
	Deleterious	54
	**Total**	**92**
PolyPhen-2	Benign	30
	Possibly damaging	15
	Probably damaging	47
	**Total**	**92**
PROVEAN	Neutral	46
	Deleterious	46
	**Total**	**92**
PANTHER	Benign	0
	Possibly damaging	50
	Probably damaging	42
	**Total**	**92**
SNAP2	Neutral	44
	Effective	48
	**Total**	**92**
PhD-SNP	Neutral	52
	Disease	40
	**Total**	**92**
Predict SNP	Neutral	45
	Deleterious	47
	**Total**	**92**

**Table 2:** The 19 high-risk missense variants of the *CYP1B1* gene that were predicted to be “damaging” or “deleterious” by all the seven pieces of software.

rs IDs	Variant	SIFT		PolyPhen 2		PROVEAN		PANTHER		SNAP2		Phd SNP	Predict SNP	
		Score	Prediction	Score	Prediction	Score	Prediction	Score	Prediction	Score	Prediction	Prediction	Score	Prediction
rs201824781	P52L	0	Deleterious	1	Probably Damaging	−6.33	Deleterious	0.85	Probably Damaging	58	effect	Disease	0.86908	Deleterious
rs28936700	G61E	0	Deleterious	1	Probably Damaging	−6.60	Deleterious	0.85	Probably Damaging	90	effect	Disease	0.86908	Deleterious
rs112014184	G90R	0	Deleterious	1	Probably Damaging	−6.94	Deleterious	0.85	Probably Damaging	91	effect	Disease	0.86908	Deleterious
rs73625096	P118L	0	Deleterious	1	Probably Damaging	−8.41	Deleterious	0.85	Probably Damaging	73	effect	Disease	0.86908	Deleterious
rs72481807	E173K	0	Deleterious	1	Probably Damaging	−3.25	Deleterious	0.78	Probably Damaging	84	effect	Disease	0.86908	Deleterious
rs72480439	D291G	0.015	Deleterious	0.985	Probably Damaging	−6.30	Deleterious	0.85	Probably Damaging	84	effect	Disease	0.86908	Deleterious
rs200182794	Y349D	0.022	Deleterious	0.917	Probably Damaging	−6.45	Deleterious	0.74	Probably Damaging	38	effect	Disease	0.86908	Deleterious
rs55771538	G365R	0.003	Deleterious	0.991	Probably Damaging	−6.24	Deleterious	0.85	Probably Damaging	72	effect	Disease	0.86908	Deleterious
rs55771538	G365W	0	Deleterious	0.998	Probably Damaging	−6.34	Deleterious	0.85	Probably Damaging	75	effect	Disease	0.86908	Deleterious
rs72480442	R368C	0	Deleterious	0.996	Probably Damaging	−7.34	Deleterious	0.85	Probably Damaging	92	effect	Disease	0.60548	Deleterious
rs79204362	R368H	0.01	Deleterious	0.978	Probably Damaging	−4.59	Deleterious	0.85	Probably Damaging	90	effect	Disease	0.86908	Deleterious
rs104893622	D374N	0.006	Deleterious	0.981	Probably Damaging	−4.74	Deleterious	0.85	Probably Damaging	59	effect	Disease	0.71871	Deleterious
rs104893629	N423Y	0.001	Deleterious	1	Probably Damaging	−7.62	Deleterious	0.85	Probably Damaging	89	effect	Disease	0.86908	Deleterious
rs201181935	D430E	0.002	Deleterious	0.922	Probably Damaging	−3.57	Deleterious	0.85	Probably Damaging	59	effect	Disease	0.86908	Deleterious
rs72481808	P442A	0.022	Deleterious	1	Probably Damaging	−7.53	Deleterious	0.85	Probably Damaging	34	effect	Disease	0.86908	Deleterious
rs72549376	R444Q	0.003	Deleterious	1	Probably Damaging	−3.77	Deleterious	0.85	Probably Damaging	69	effect	Disease	0.86908	Deleterious
rs148233007	F445L	0	Deleterious	1	Probably Damaging	−5.66	Deleterious	0.85	Probably Damaging	93	effect	Disease	0.86908	Deleterious
rs28936701	R469W	0	Deleterious	1	Probably Damaging	−7.28	Deleterious	0.74	Probably Damaging	98	effect	Disease	0.60697	Deleterious
rs104894979	C470Y	0	Deleterious	1	Probably Damaging	−10.10	Deleterious	0.85	Probably Damaging	89	effect	Disease	0.86908	Deleterious

### Effect of variations on CYP1B1 protein stability

Two biological tools, namely I-Mutant 2.0 and MUpro, were independently used to investigate the effects of all the 19 missense variations on the CYP1B1 protein stability. The I-Mutant 2.0 server predicted the effect of amino acid change in the form of protein stability Gibb’s free energy change (DDG) and calculated the RI of the variations, while MUpro predicted the effect of protein stability for single site mutation in the form of Gibb’s free energy change (DDG). Of the 19 high-risk missense variations identified, I-Mutant 2.0 revealed that all amino acid variations profoundly affected the CYP1B1 protein by decreasing its stability, except for four variants: G61E, P118L, Y349D, and D430E, which did not influence the protein stability. Apart from I-Mutant 2.0, MUpro identified only P52L had no effect on protein stability and the remaining exhibited a reduction in protein stability. The details of I-Mutant 2.0 and MUpro analysis are provided in Table 3 and Table S2.

### Conservancy analysis of high-risk variations and secondary structure of CY1B1

**Figure 1:**
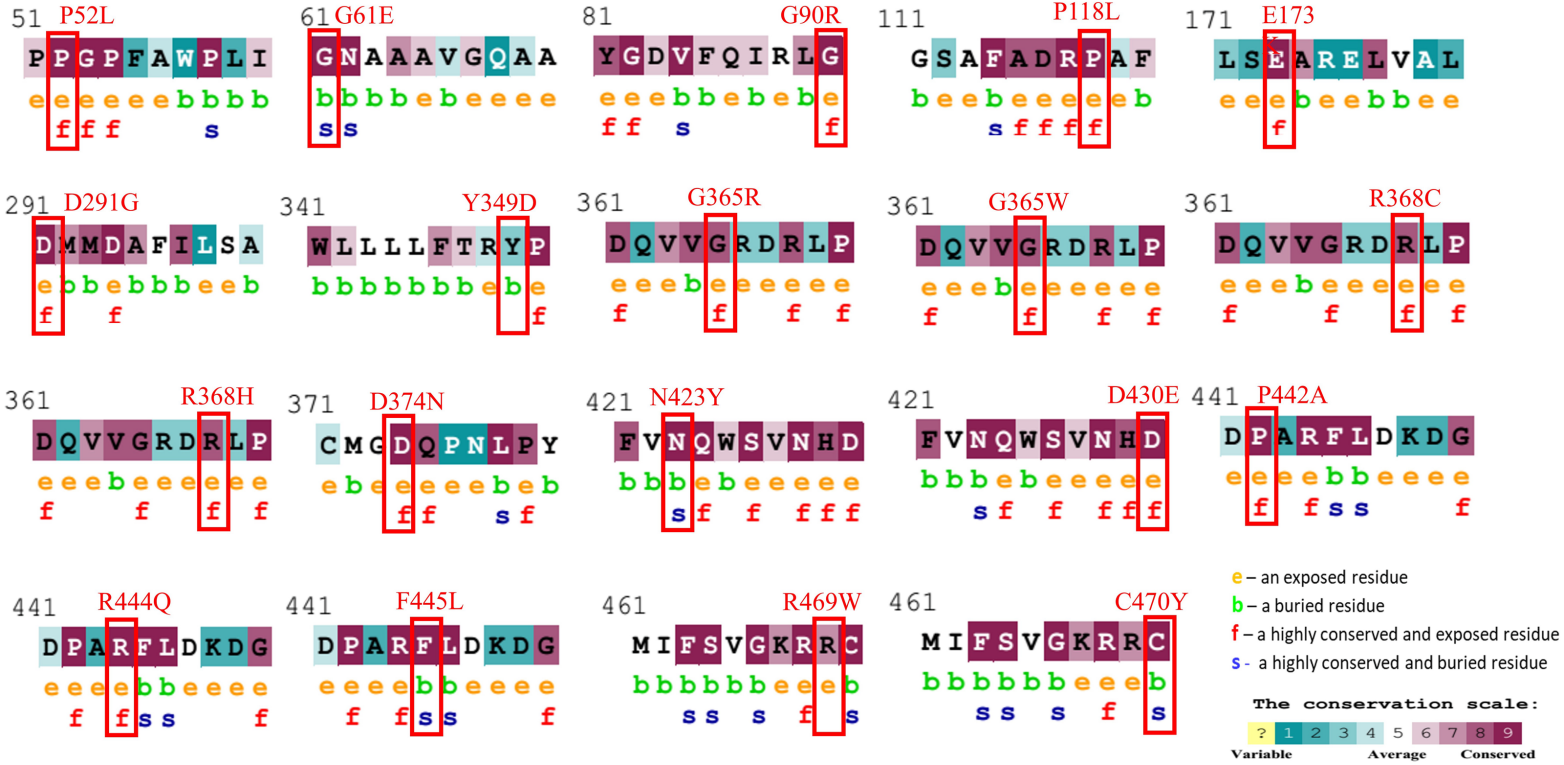
Evolutionary conservancy results of CYP1B1 high-risk missense variants predicted by the ConSurf server. The red boxes indicate the positions of wild-type amino acids that would be affected by the missense change. In addition, the tool depicted the conservation scores that range from 1 to 9, where conserved sites get scores of 7 to 9, and the characteristics of residue are either exposed (e), buried (b), highly conserved and exposed (functional, f), or highly conserved and buried (s).

The evolutionary conservation characteristic of all the common 19 missense variants was accomplished by the Consurf server. It is essential to determine the magnitude of an amino acid substitution to alter the protein’s structure and function. Results revealed that all amino acid variations occurred in the highly conserved region of the CYP1B1 protein except for one substitution (Y349D) that was observed in the variable region (Fig. 1). Additionally, the solvent accessibility feature predicted that 13 variants (P52L, G90R, P118L, E173K, D291G, G365W, G365R, R368H, R368C, D374N, D430E, P442A, and R444Q) were found to be highly conserved and exposed residues, four variants (G61E, N423Y, F445L, and C470Y) were observed to be highly conserved and buried residues and two variants (Y349D and R469W) were identified as buried and exposed residues. The evolutionary conservation and solvent accessibility feature of all the 19 substitutions are demonstrated in (Fig. 1). Generally, it has been considered that exposed residues have an impact on protein function and, thereby, highly conserved and exposed residues are recognized to have functional importance. On the other hand, buried residues are incorporated into the protein structure maintenance, and thus highly conserved and buried residues reveal their structural importance (Zhang et al., 2017). Based on this analysis, it can be concluded that changes in 19 regions of the CYP1B1 protein, which play an important role in the structural and functional behavior of protein integrals, confirm their deleterious nature.

Furthermore, of the total 543 amino acids, 255 amino acids (46.96%) were found in the alpha helix, 207 amino acids (38.12%) in the random coils region, 61 (11.23%) in the extended strand, and 20 (3.68%) in the beta turns of the CYP1B1 protein as predicted by SOPMA (Fig. S1).

### Structural examination of native and mutants of *CYP1B1*

**Figure 2:**
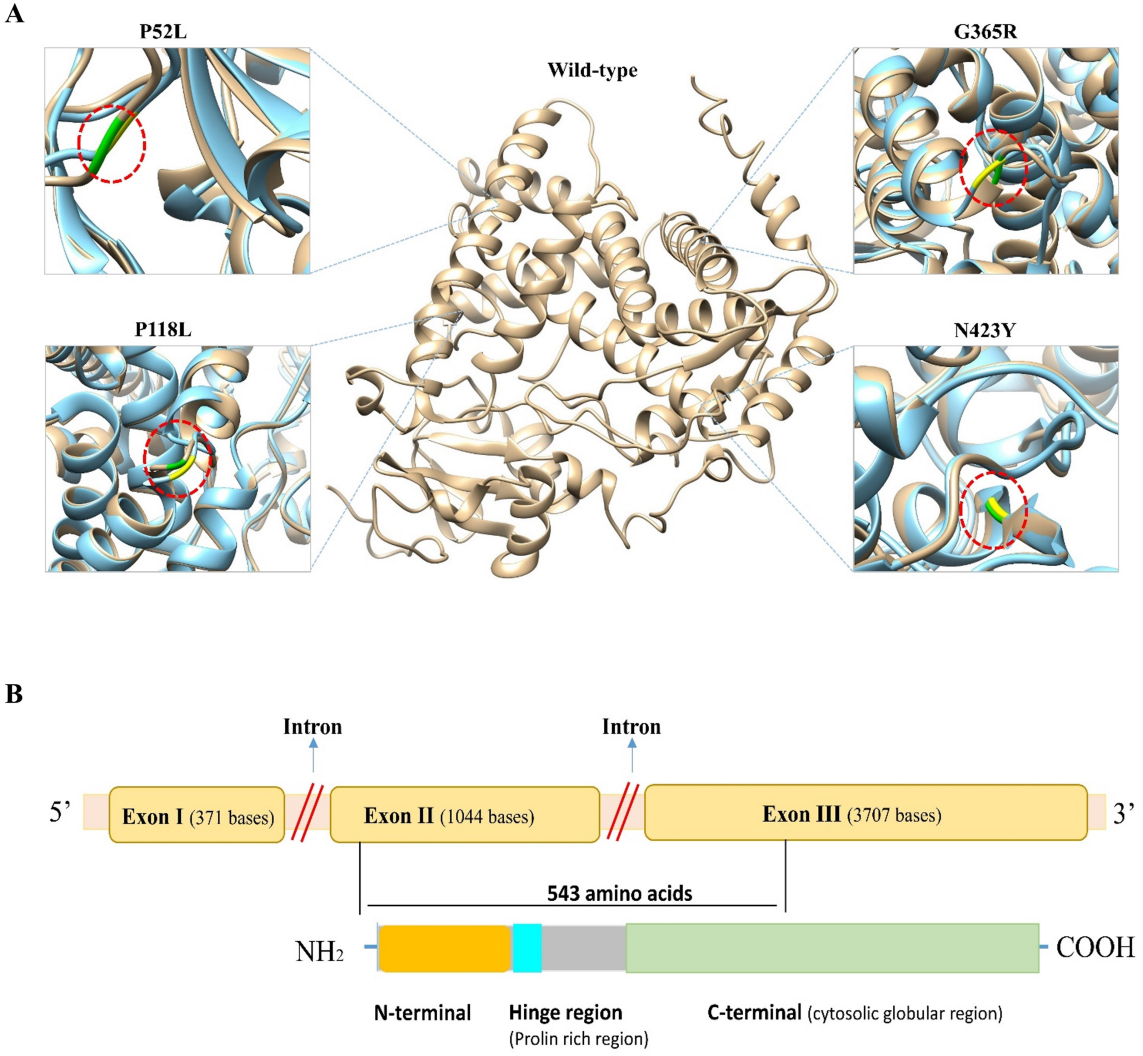
Three-dimensional (3D) structural comparison of wild-type and mutants of CYP1B1 protein. (A) The wild-type structure is shown in light brown and mutant in light blue colour. The wild-type amino acid is labelled in green, and the mutant substitution is in yellow in the red circular diagrams. (B) A schematic structural diagram of CYP1B1 based on published literature.

To predict the structural impact of these 19 high-risk variations, first the 3D structure of the CYP1B1 protein was obtained from the PDB database (PDB ID: 3PM0). The structure had some missing residues; therefore, to generate the complete structure of the CYP1B1 protein, the I-TASSER server was utilized. From the generated models of I-TASSER, the best model with a C-score of 0.16, an estimated TM-score of 0.73 ± 0.11, and an estimated RMSD of 7.0 ± 4.1 A was selected and subjected to further evaluation. The quality of the best generated model structure was validated through the ERRAT and VERIFY3D tools of the SAVES server. The ERRAT tool provided the overall quality score, which was 92.57. The VERIFY3D tool estimates the compatibility of a protein model based on local statistical potentials and predicts that the CYP1B1 protein model passed the criteria with an average score of 89.94% and is worth further investigation. Similarly, the 3D structures of all 19 mutants of CYP1B1 were generated, and their graphical illustration and superimposition with the wild-type structure were performed by UCSF Chimera software version 1.14. The 3D wild-type and some mutant structures are depicted in Fig. 2A. In addition, the RMSD of all 19 mutant structures was calculated using the TM-Align server, and their results are shown in **Table 3**. The results of the TM-Align server elucidated that all 19 mutant structures have profound impacts on the CYP1B1 protein. This suggests that when these variations occur in the CYP1B1 protein, they may interfere with its functional behavior.

**Table 3:** The results show the effects on protein stability and TM-align score of 19 high-risk missense variations of the CYP1B1.

rs ID	Variant	I-Mutant 2.0		MUpro		TM Align		gnomAD	Population
		DDG	Stability	DDG	Stability	RMSD	TM score	Allele frequency	
rs201824781	P52L	0.59	Decrease	0.470	Increase	1.47	0.971	0.000299/42	Ashkenazi Jewish, European, Latino, African, others
rs28936700	G61E	−0.2	Increase	−0.133	Decrease	1.2	0.984	0.000171/24	European, South Asian, Latino, others
rs112014184	G90R	−1.56	Decrease	−0.606	Decrease	0.98	0.989	Not available	Not available
rs73625096	P118L	0.68	Increase	−0.123	Decrease	1.53	0.978	0.000005/1	Latino
rs72481807	E173K	−0.24	Decrease	−0.998	Decrease	1.3	0.980	0.000007/1	Others
rs72480439	D291G	−1.26	Decrease	−2.096	Decrease	1.34	0.980	0.000008/2	East Asian
rs200182794	Y349D	−0.03	Increase	−1.239	Decrease	1.26	0.985	0.000008/2	African, European (non-Finnish)
rs55771538	G365R	−1.02	Decrease	−0.502	Decrease	1.38	0.979	0.000004/1	European (non-Finnish)
rs55771538	G365W	−0.66	Decrease	−0.606	Decrease	1.49	0.978	0.000004/1	European (non-Finnish)
rs72480442	R368C	−0.8	Decrease	−0.59	Decrease	1.22	0.979	0.000207/29	Latino. European (non-Finnish), others
rs79204362	R368H	−1.53	Decrease	−0.708	Decrease	1.34	0.978	0.001248/175	South Asian, East Asian, Ashkenazi Jewish, Latino, European, African
rs104893622	D374N	−0.74	Decrease	−0.62	Decrease	1.27	0.979	Not available	Not available
rs104893629	N423Y	0.02	Decrease	−0.750	Decrease	1.4	0.979	0.000007/1	European (non-Finnish)
rs201181935	D430E	−0.46	Increase	−0.919	Decrease	1.31	0.978	0.000007/1	East Asian
rs72481808	P442A	−1.07	Decrease	−0.72	Decrease	0.68	0.993	Not available	Not available
rs72549376	R444Q	−1.39	Decrease	−1.35	Decrease	1.41	0.977	0.000007/1	European (non-Finnish)
rs148233007	F445L	−2.41	Decrease	−0.856	Decrease	1.37	0.982	0.000007/1	South Asian, European (non-Finnish)
rs28936701	R469W	−0.75	Decrease	−0.577	Decrease	1.38	0.977	0.000036/5	South Asian, East Asian, European (non-Finnish), Latino
rs104894979	C470Y	0.33	Decrease	−1.111	Decrease	1.42	0.977	Not available	Not available

### Molecular dynamics simulation (MDS) analysis

**Figure 3:**
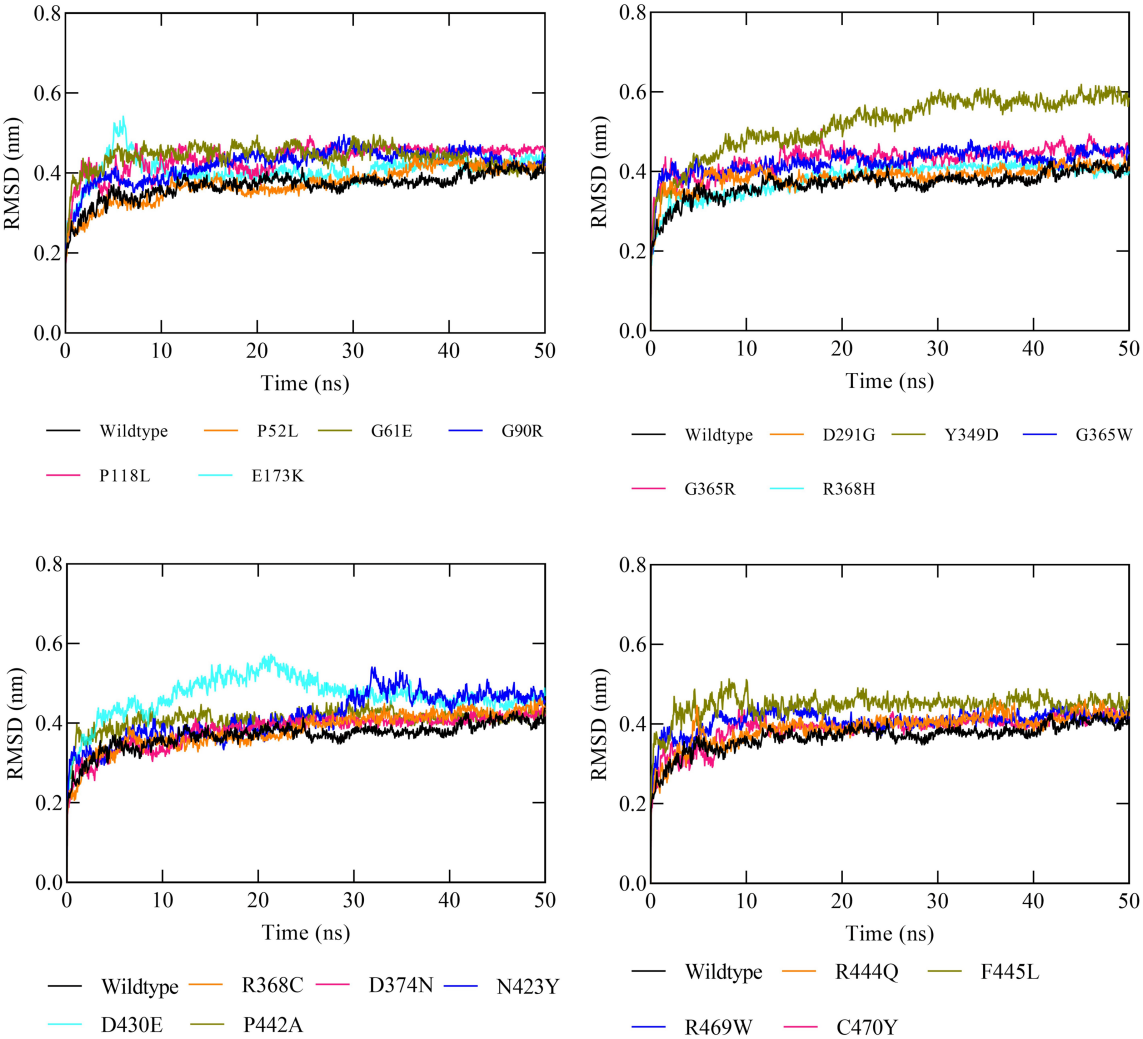
The RMSD results of all the 19 high-risk missense variants of CYP1B1 after the 50 ns MD simulation period.

The molecular dynamic simulation (MDS) was executed to evaluate the protein stability following a missense variation in the CYP1B1 protein. The MDS was run up to 50 ns and the RMSD values were noted for the wild-type and for all the 19 high-risk missense variations. Results demonstrated that the CYP1B1 wild-type structure showed an RMDS in the range of 0.36 nm and structural stability throughout the simulation period (Fig. 3). These results indicate that the native structure attained a stable conformation in the MDS period. Overall, the RMSD value was observed to be higher in all the 19 mutants. Six variants (P52L, D291G, R368H, R368C, D374N, and C470Y) demonstrated an RMSD ∼0.38 nm. The highest value of RMSD was observed in three variants (Y349D, D430E, and F445L) that had ∼0.52, 0.46, and 0.44 nm, respectively, compared to the wild-type protein structure. The remaining variants’ RMSD was observed to be greater than 0.40 nm. Figure 3 depicts the RMSD results of all 19 variants after the MDS run. Generally, the higher the RMSD value indicates, the higher the deviation of the mutant with respect to the native protein structure (Agrahari et al., 2019). Therefore, the results of MDS demonstrated that these variants were predicted to destabilize the native protein structure, which could interfere with the normal functioning of the CYP1B1 protein.

### Investigation of CYP1B1-interacting candidates

**Figure 4:**
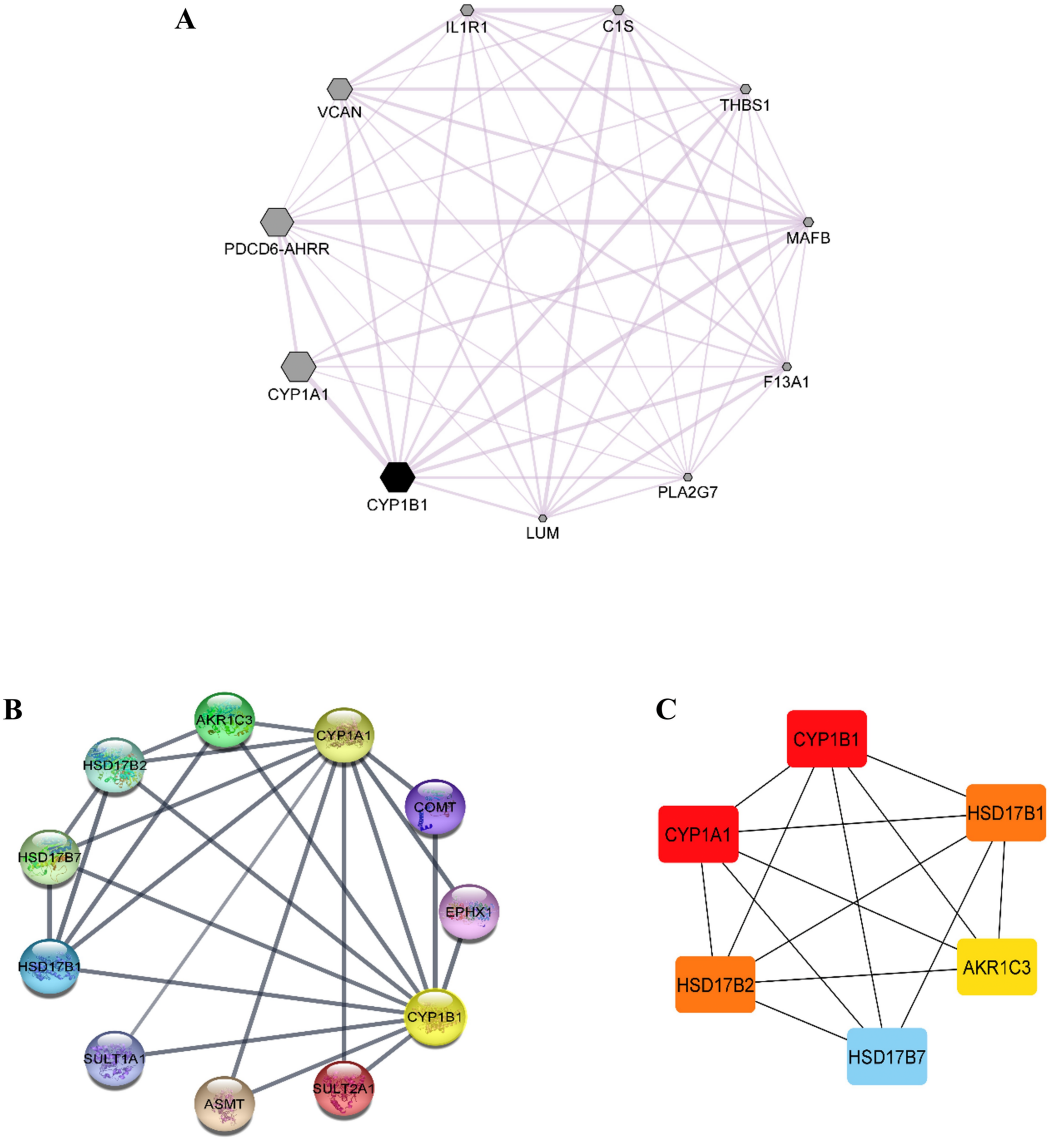
Co-expression and protein-protein interaction (PPI) of CYP1B1. (A) Co-expression network of CYP1B1 constructed by GeneMANIA plugin of Cytoscape. In the constructed network, the size of the node represents the degree of co-expression. The higher the degree of co-expression, the bigger the size of the node. (B) The protein-protein interaction (PPI) network of CYP1B1. (C) A network of hub candidates predicted to be highly interacted with CYP1B1 by the cytoHubba plugin Cytoscape software.

Co-expression data for the *CYP1B1* gene was obtained from the GeneMANIA database and then imported into the Cytoscape software to build a network based on the co-expression score (Fig. 4A). Results elucidate that *CYP1B1* was highly co-expressed with *CYP1A1,* followed by *PDCD6-AHRR* and *VCAN* genes, respectively. The least expression of *CYP1B1* was observed with the *LUM*, *PLA2G7*, and *F13A1* genes. Furthermore, the protein-protein interaction (PPI) network was also generated *via* cytoscape software (Fig. 4B). Then, the cytoHubba plugin was employed to identify the highly interacting proteins with CYP1B1. Through cytoHubba, it was discovered that five candidates, which were: CYP1A1, HSD17B1, HSD17B2, AKR1C3, and HSD17B7, demonstrated close interaction with CYP1B1 protein (Fig. 4C).

### Gene ontology (GO) and pathway enrichment analysis of CYP1B1

**Figure 5:**
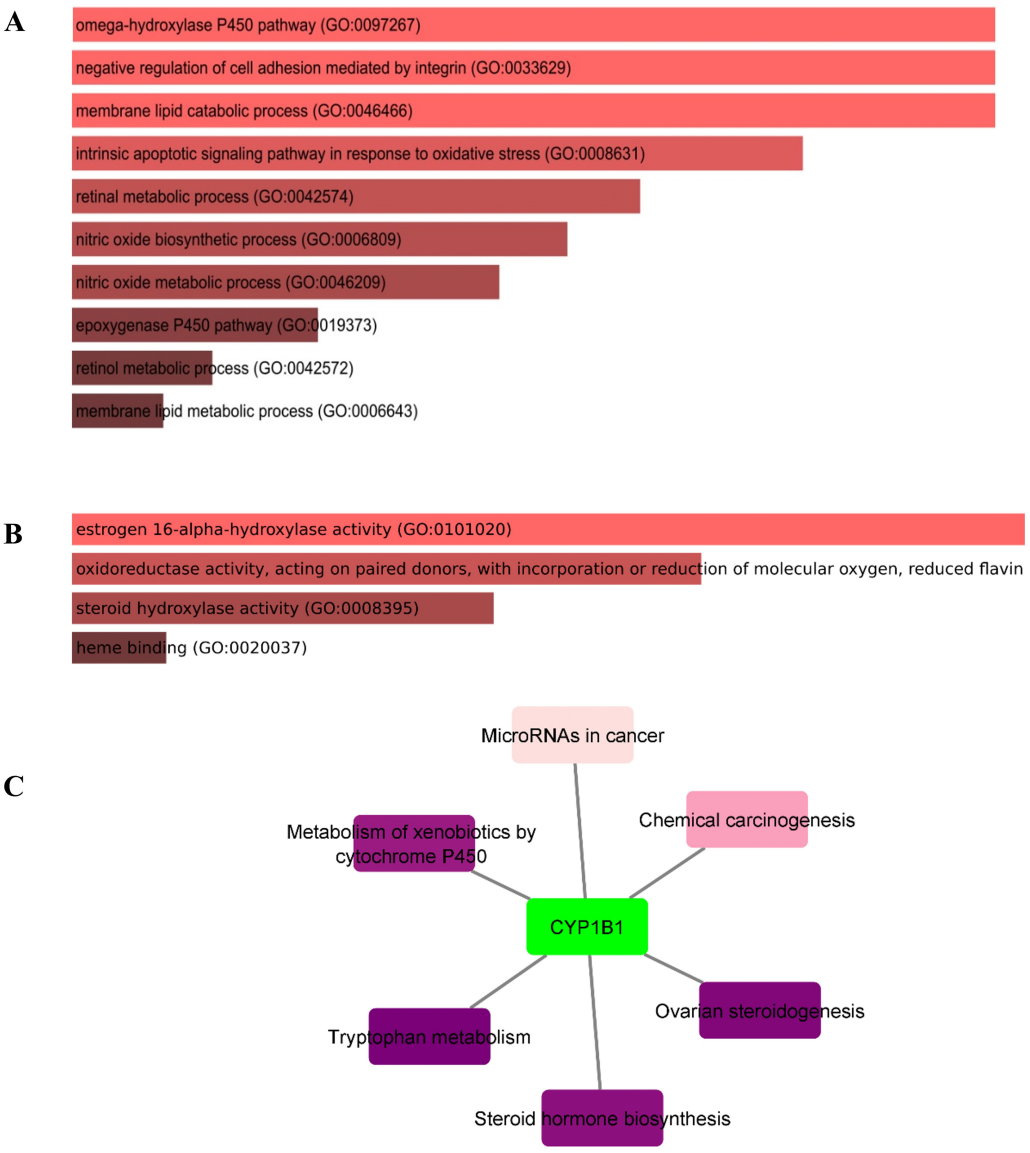
Gene ontology (GO) and pathways analyses of CYP1B1. The most enriched; (A) biological processes, (B) molecular functions, and (C) KEGG pathways of the CYP1B1.

In this study, the Enricher server was utilized for Gene Ontology (GO) and pathway analyses. In total, 59 biological processes (BPs) were discovered for CYP1B1 as documented in Table S3. The *p-value* for all 59 BPs was less than 0.05, indicating that CYP1B1 plays an important biological role in these processes. Fig. 5A illustrates the top ten BPs. Subsequently, only four molecular functions were discovered, which were estrogen 16-alpha-hydroxylase activity, oxidoreductase activity, steroid hydroxylase activity, and heme binding, respectively (Fig. 5B). Likewise, the cellular component (CC) was widely observed in the endoplasmic reticulum membrane and intracellular membrane-bounded organelles. The detailed results of GO are tabulated in Table S3. Moreover, six KEGG pathways were discovered for CYP1B1, which were: Tryptophan metabolism, ovarian steroidogenesis, steroid hormone biosynthesis, metabolism of xenobiotics by cytochrome P450, chemical carcinogenesis, and microRNAs in cancer Fig. 5C Table S4).

## Discussion

The implementation of high-throughput sequencing technology led to the oversaturation of nsSNPs that has recently exponentially increased in human genome databases. In terms of their related contribution to the specific phenotype, characterization of any nsSNP is a cost-effective, cumbersome, and time-consuming procedure, making it hard to evaluate the biological impact of these variations through laboratory trials. In contrast, employing computational techniques assists in filtering out the pathogenic or disease-associated variants from neutrals from a large pool of SNP datasets. Among the two causative genes (*CYP1B1* and *LTBP2*) of primary congenital glaucoma (PCG), *CYP1B1* is found to be highly mutated and responsible for the etiology of PCG patients worldwide (Firasat et al., 2008). Therefore, a comprehensive systematic approach was carried out to evaluate the impacts of missense variations at functional and structural levels.

After employing comprehensive functional analysis, 19 missense variants (P52L, G61E, G90R, P118L, E173K, D291G, Y349D, G365W, G365R, R368H, R368C, D374N, N423Y, D430E, P442A, R444Q, F445L, R469W, and C470Y) were found to be common and were considered the high-risk deleterious variants of the CYP1B1 gene (Table 2). At position 52, the proline amino acid residue was replaced by leucine, a bigger residue than the wild-type. Prolines are known to have very rigid structures, and any substitution with proline could disturb the local structure of the protein. This variant was previously discovered by Campos-Mollo et al. (2009). They carried out a functional analysis study by transiently transfected human embryonic kidney 293T (HEK-293-T) cells and found that the P52L variant reduced the catalytic activity of CYP1B1 and imparted high protein instability (Campos-Mollo et al., 2009). Conservancy analysis revealed that this variation occurred in the highly conserved and exposed region of the protein, highlighting its magnitude of penetrance in malfunctioning of the protein (Fig. 1). The stability and MDS analysis also demonstrated the role in the reduction of protein stability and RMSD deviation (0.37 nm) of this variant protein compared with the native protein (Fig. 3). Likewise, at position 61, the hinge region, where glycine, which is a neutral amino acid, was substituted by a negatively charged glutamate. This variant (G61E) was identified in many populations, as reported in previous studies (Afzal et al., 2019; El-Ashry, El-Aziz & Bhattacharya, 2007; El-Gayar et al., 2009; Jubair et al., 2019; Stoilov et al., 1998). In this study, it has been shown that most of the high-risk missense variations are found in the carboxyl terminal region of the CYP1B1 protein, which is incorporated into several catabolic activities. This variation was found to be in the highly conserved and buried region and showed a high RMSD deviation value (0.43 nm) compared to the wild-type protein (Fig. 3). The mutated residue introduced a negative charge, which may cause repulsion of other residues with the same charge and may interfere with the protein’s functional activity or incorrect conformation of the protein structure.

Two variants (G90R and G365R) where glycine, a neutral amino acid, was mutated by a positively charged arginine, exhibited instability as well as high RMSD (0.42 and 0.43 nm, respectively), which could affect the proper protein folding (Fig. 3). Whereas, three variants (R368C, R444Q, and R469W) in which a positively charged amino acid (Arginine) was replaced by three neutral amino acids (Cysteine, Glutamine, and Tryptophan) at positions 368, 444, and 469, respectively. The substituted residues are bigger in size and change the charge of the native residue, which may lead to protein folding problems and will disturb the local structure (Shahid et al., 2022). R368C and R444Q existed in the highly conserved and exposed region, and R469W was in the buried region of the protein, influencing the reduction in stability and showing an RMSD of around 0.40 nm (Figs. 1 and 3). The frequency of these three variants is very high, as they were identified in various populations (Table 3) (Faiq et al., 2013; Li et al., 2011; Stoilov et al., 1998). Similarly, the remaining high-risk missense variations (P118L, E173K, D291G, Y349D, G365W, R368H, D374N, N423Y, D430E, P442A, F445L, and C470Y) were found to be distributed in many populations as discovered by various research studies (Bejjani et al., 2000; Chen et al., 2015; Colomb, Kaplan & Garchon, 2003; El-Gayar et al., 2009; Li et al., 2011; Stoilov et al., 1998). The findings suggest that these variants may influence the protein structure and functions, either by misfolding the local structure or resulting in the loss of protein–protein interactions of the CYP1B1 protein. Generally, the higher the RMSD value indicates, the higher the deviation of the mutant with respect to the native protein structure (Agrahari et al., 2019). Therefore, the results of MDS demonstrated that these variants were predicted to destabilize the native protein structure, which could interfere with the normal functioning of the CYP1B1 protein.

In humans, the *CYP1B1* gene comprises three exons with 371, 1,044 and 3,707 base pairs in length, respectively, that encode a protein with a length of 543 amino acids. It has been seen that the coding region for the CYP1B1 protein starts from the 5′ end of exon number two and ends within the third exon (Faiq et al., 2013). This protein has three structural regions: N-terminal membrane-bound region of 53 residues; a hinge region comprised of 10 proline-rich amino acid residues that participate in the flexibility of the protein; and a 480 amino acid long C-terminal harboring a heme-binding region (Vasiliou & Gonzalez, 2008). The C-terminal is a highly conserved cytosolic globular domain that is essential for the normal function of this class of enzymes. A schematic diagram of CYP1B1 based on a literature review has been shown in Fig. 2B.

Co-expression analysis demonstrated that *CYP1B1* was highly co-expressed with *CYP1A1,* followed by *PDCD6-AHRR* and *VCAN* genes, respectively (Fig. 4A). The *CYP1A1* also belongs to the same superfamily of cytochrome P450 and its encoded product performs similar functions as the CYP1B1, *i.e.,* detoxification of xenobiotics, drug metabolism, synthesis of cholesterol, steroids, and other lipids (Kyoreva et al., 2021). The *VCAN* is a member of the versican proteoglycan family. The encoded product is incorporated into cell proliferation, migration, adhesion, and angiogenesis. It also plays a central role in tissue morphogenesis and maintenance (Wight, 2002). However, these results delineate the additional functions of the *CYP1B1* gene by being co-expressed with these genes. The PPI network analysis discovered that five candidates, which were: CYP1A1, HSD17B1, HSD17B2, AKR1C3, and HSD17B7, exhibited close interaction with CYP1B1 protein (Figs. 4B and 4C). The CYP1A1 protein is a member of the CYP protein family, which plays a significant role in the activation of compounds with carcinogenic properties (30). AKR1C3 (Aldo-keto reductase family 1 member C3) is an enzyme that catalyses the conversion of aldehydes and ketones into their respective alcohols. This enzyme is one of the promising biomarkers for the development of prostate cancer (29). HSD17B1, HSD17B2, and HSD17B7 are the members of the 17 β-hydroxysteroid dehydrogenase family that are incorporated in the end phase of the biosynthesis of active steroid hormones. HSD17B1 and HSD17B7 are reductive enzymatic proteins, while HSD17B2 is an oxidative enzymatic protein (Lukacik, Kavanagh & Oppermann, 2006). The findings of this study suggest that CYP1B1 might have many functions by interacting with CYP1A1, HSD17B1, HSD17B2, AKR1C3, and HSD17B7 proteins.

The GO analysis revealed that CYP1B1 is predominantly involved in the omega-hydroxylase P450 pathway, negative regulation of cell adhesion mediated by integrin, membrane lipid catabolic process, intrinsic apoptotic signaling pathway in response to oxidative stress, retinal metabolic process, and many other biological processes (Table S3). Figure 5A illustrates the top ten BPs. CYP1B1 was discovered to have six KEGG pathways, which were: Tryptophan metabolism, ovarian steroidogenesis, steroid hormone biosynthesis, metabolism of xenobiotics by cytochrome P450, chemical carcinogenesis, and microRNAs in cancer (Fig. 5C). All these findings were consistent with the earlier research studies and suggest that CYP1B1 is a multifunctional protein and is incorporated into the regulation of multiple pathways.

However, there were several admitted confines to this study, such as that it was an *in silico* study where publically available data was utilized for the analysis. We employed a comparative approach to improve the accuracy and enhance the reliability, but there were some pitfalls in the study as these were the computational tools that used different algorithms. These tools possibly provide some false negative or positive outcomes due to overlapping concepts in their predictions that restrict us from being fully confident unless verified through wet lab experiments. Moreover, although most of the predicted variants have been reported in various research studies, experimental validation is still required to affirm the actual consequences. Functional assays, segregation analysis, genetic studies, and epidemiological data could be implemented to predict the pathogenicity and improve the classification accuracy, respectively. Despite these limitations, the study presented here to some extent overcomes them as it was a comprehensive *in silico* study where molecular dynamic simulation was also performed to enhance the reliability of the results, thereby providing a quick, critical, and cost-effective approach to evaluate the mutational spectra of the *CYP1B1* gene and to discover the probable biomarkers that can be helpful for the understanding and management of primary congenital glaucoma.

## Conclusion

The present study was focused on identifying the high-risk missense deleterious variants of the CYP1B1 gene that are associated with primary congenital glaucoma (PCG) through comparative *in silico* approaches. A spectra of 19 high-risk missense deleterious variations, which were primarily involved in PCG pathogenesis, were identified from all of the retrieved data. Furthermore, phylogenetic analysis, molecular dynamic simulation results, and structural analysis of the wild-type and mutant forms revealed that the majority of the variants were in highly conserved regions and could reduce protein stability, potentially causing CYP1B1 protein dysfunction. Moreover, the results of the gene expression, protein-protein interaction, gene ontology, and pathway analysis were mostly consistent with the earlier research studies. Nevertheless, this study provides an essential profile of *CYP1B1* and its highly associated missense variants with the PCG that can be potentially used as diagnostic markers and will be helpful for the management of primary congenital glaucoma studies.

##  Supplemental Information

10.7717/peerj.14132/supp-1 Supplemental Information 1 CYP1B1 missense variations detailed data

10.7717/peerj.14132/supp-2 Supplemental Information 2 CYP1B1 protein stability results derived from I-Mutant and MUpro web tools

10.7717/peerj.14132/supp-3 Supplemental Information 3 CYP1B1 gene ontology (GO) analysis data

10.7717/peerj.14132/supp-4 Supplemental Information 4 CYP1B1 KEGG pathways analysis data

10.7717/peerj.14132/supp-5 Supplemental Information 5 Detailed information of secondary structure of CYP1B1

10.7717/peerj.14132/supp-6 Supplemental Information 6 Molecular Dynamics Simulation raw data (replicate 1) The XVG files can be opened by using Gromacs Software plugins (https://www.gromacs.org/).

10.7717/peerj.14132/supp-7 Supplemental Information 7 Molecular Dynamics Simulation Raw data (replicate 2) The XVG files can be opened by using Gromacs Software plugins (https://www.gromacs.org/).

## Additional Information and Declarations

### Data Availability

The following information was supplied regarding data availability: The raw data are available in the Supplemental Files.

## References

[ref-1] Adzhubei IA, Schmidt S, Peshkin L, Ramensky VE, Gerasimova A, Bork P, Kondrashov AS, Sunyaev SR (2010). A method and server for predicting damaging missense mutations. Nature Methods.

[ref-2] Afzal R, Firasat S, Kaul H, Ahmed B, Siddiqui SN, Zafar SN, Shahzadi M, Afshan KJCA (2019). Mutational analysis of the CYP1B1 gene in Pakistani primary congenital glaucoma patients: identification of four known and a novel causative variant at the 3′ splice acceptor site of intron 2. Congenital Anomalies.

[ref-3] Agrahari AK, Krishna Priya M, Praveen Kumar M, Tayubi IA, Siva R, Prabhu Christopher B, George Priya Doss C, Zayed H (2019). Understanding the structure-function relationship of HPRT1 missense mutations in association with Lesch-Nyhan disease and HPRT1-related gout by in silico mutational analysis. Computers in Biology and Medicine.

[ref-4] Akarsu AN, Turacli ME, Aktan SG, Barsoum-Homsy M, Chevrette L, Sayli BS, Sarfarazi M (1996). A second locus (GLC3B) for primary congenital glaucoma (Buphthalmos) maps to the 1p36 region. Human Molecular Genetics.

[ref-5] Ali M, McKibbin M, Booth A, Parry DA, Jain P, Riazuddin SA, Hejtmancik JF, Khan SN, Firasat S, Shires M, Gilmour DF, Towns K, Murphy AL, Azmanov D, Tournev I, Cherninkova S, Jafri H, Raashid Y, Toomes C, Craig J, Mackey DA, Kalaydjieva L, Riazuddin S, Inglehearn CF (2009). Null mutations in LTBP2 cause primary congenital glaucoma. American Journal of Human Genetics.

[ref-6] Ashkenazy H, Erez E, Martz E, Pupko T, Ben-Tal N (2010). ConSurf 2010: calculating evolutionary conservation in sequence and structure of proteins and nucleic acids. Nucleic Acids Research.

[ref-7] Azfaralariff A, Farahfaiqah F, Shahid M, Sanusi SA, Law D, Isa ARM, Muhamad M, Tsui TT, Fazry SJJoE (2022). Marantodes pumilum: systematic computational approach to identify their therapeutic potential and effectiveness. Journal of Ethnopharmacology.

[ref-8] Badawi AH, Al-Muhaylib AA, Owaifeer AMAl, Al-Essa RS, Al-Shahwan SA (2019). Primary congenital glaucoma: an updated review. Saudi Journal of Ophthalmology.

[ref-9] Bejjani BA, Stockton DW, Lewis RA, Tomey KF, Dueker DK, Jabak M, Astle WF, Lupski JR (2000). Multiple CYP1B1 mutations and incomplete penetrance in an inbred population segregating primary congenital glaucoma suggest frequent de novo events and a dominant modifier locus. Human Molecular Genetics.

[ref-10] Bekker H, Berendsen H, Dijkstra E, Achterop S, Vondrumen R, Vanderspoel D, Sijbers A, Keegstra H, Renardus M (1993). Gromacs-a parallel computer for molecular-dynamics simulations.

[ref-11] Bendl J, Stourac J, Salanda O, Pavelka A, Wieben ED, Zendulka J, Brezovsky J, Damborsky J (2014). PredictSNP: robust and accurate consensus classifier for prediction of disease-related mutations. PLOS Computational Biology.

[ref-12] Bhattacharya S, Ray SJG (2021). In silico screening and exploration into phenotypic alterations of deleterious oncogenic single nucleotide polymorphisms in HSPB1 gene. Genomics.

[ref-13] Bromberg Y, Rost B (2007). SNAP: predict effect of non-synonymous polymorphisms on function. Nucleic Acids Research.

[ref-14] Campos-Mollo E, Lopez-Garrido MP, Blanco-Marchite C, Garcia-Feijoo J, Peralta J, Belmonte-Martinez J, Ayuso C, Escribano J (2009). CYP1B1 mutations in Spanish patients with primary congenital glaucoma: phenotypic and functional variability. Molecular Vision.

[ref-15] Capriotti E, Calabrese R, Casadio R (2006). Predicting the insurgence of human genetic diseases associated to single point protein mutations with support vector machines and evolutionary information. Bioinformatics.

[ref-16] Capriotti E, Fariselli P, Casadio RJNar (2005). I-Mutant2.0: predicting stability changes upon mutation from the protein sequence or structure. Nucleic Acids Research.

[ref-17] Chen EY, Tan CM, Kou Y, Duan Q, Wang Z, Meirelles GV, Clark NR, Ma’ayan A (2013). Enrichr: interactive and collaborative HTML5 gene list enrichment analysis tool. BMC Bioinformatics.

[ref-18] Chen L, Huang L, Zeng A, He J (2015). CYP1B1 gene mutations with incomplete penetrance in a Chinese pedigree with primary congenital glaucoma: a case report and review of literatures. International Journal of Clinical and Experimental Medicine.

[ref-19] Cheng J, Randall A, Baldi P (2006). Prediction of protein stability changes for single-site mutations using support vector machines. Proteins.

[ref-20] Choi Y, Sims GE, Murphy S, Miller JR, Chan AP (2012). Predicting the functional effect of amino acid substitutions and indels. PLOS ONE.

[ref-21] Colomb E, Kaplan J, Garchon HJ (2003). Novel cytochrome P450 1B1 (CYP1B1) mutations in patients with primary congenital glaucoma in France. Human Mutation.

[ref-22] Earl J, Greenhalf W, Grützmann R, Pilarsky C (2009). Single-nucleotide polymorphism (SNP) analysis to associate cancer risk. Cancer Gene Profiling. Methods in Molecular Biology.

[ref-23] El-Ashry MF, Abd El-Aziz MM, Bhattacharya SS (2007). A clinical and molecular genetic study of Egyptian and Saudi Arabian patients with primary congenital glaucoma (PCG). Journal of Glaucoma.

[ref-24] El-Gayar S, Ganesh A, Chavarria-Soley G, Al-Zuhaibi S, Al-Mjeni R, Raeburn S, Bialasiewicz AA (2009). Molecular analysis of CYP1B1 in Omani patients with primary congenital glaucoma: a pilot study. Molecular Vision.

[ref-25] Faiq M, Sharma R, Dada R, Mohanty K, Saluja D, Dada TJJocgp (2013). Genetic, biochemical and clinical insights into primary congenital glaucoma. Journal of Current Glaucoma Practice.

[ref-26] Falero-Perez J, Song YS, Sorenson CM, Sheibani N (2018). CYP1B1: a key regulator of redox homeostasis. Trends in Cell & Molecular Biology.

[ref-27] Firasat S, Riazuddin SA, Hejtmancik JF, Riazuddin S (2008). Primary congenital glaucoma localizes to chromosome 14q24.2-24.3 in two consanguineous Pakistani families. Molecular Vision.

[ref-28] Gencik A, Gencikova A, Ferak V (1982). Population genetical aspects of primary congenital glaucoma. I. Incidence, prevalence, gene frequency, and age of onset. Human Genetics.

[ref-29] Geourjon C, Deleage G (1995). SOPMA: significant improvements in protein secondary structure prediction by consensus prediction from multiple alignments. Computer Applications in the Biosciences.

[ref-30] Gudmundsson S, Singer-Berk M, Watts NA, Phu W, Goodrich JK, Solomonson M, Consortium GAD, Rehm HL, MacArthur DG, O’Donnell-Luria A (2022). Variant interpretation using population databases: lessons from gnomAD. Human Mutation.

[ref-31] Jubair S, NA-Ri SH, MA-S AN, Jabbar Suleiman AA (2019). Investigation of CYP1B1 gene involvement in primary congenital glaucoma in Iraqi children. Middle East African Journal of Ophthalmology.

[ref-32] Khalid Z, Almaghrabi O (2020). Mutational analysis on predicting the impact of high-risk SNPs in human secretary phospholipase A2 receptor (PLA2R1). Scientific Reports.

[ref-33] Kyoreva M, Li Y, Hoosenally M, Hardman-Smart J, Morrison K, Tosi I, Tolaini M, Barinaga G, Stockinger B, Mrowietz U, Nestle FO, Smith CH, Barker JN, Di Meglio P (2021). CYP1A1 enzymatic activity influences skin inflammation via regulation of the AHR pathway. The Journal of Investigative Dermatology.

[ref-34] Li N, Zhou Y, Du L, Wei M, Chen X (2011). Overview of Cytochrome P450 1B1 gene mutations in patients with primary congenital glaucoma. Experimental Eye Research.

[ref-35] Lukacik P, Kavanagh KL, Oppermann U (2006). Structure and function of human 17beta-hydroxysteroid dehydrogenases. Molecular and Cellular Endocrinology.

[ref-36] Muskhelishvili L, Thompson PA, Kusewitt DF, Wang C, Kadlubar FF (2001). In situ hybridization and immunohistochemical analysis of cytochrome P450 1B1 expression in human normal tissues. The Journal of Histochemistry and Cytochemistry.

[ref-37] Ng PC, Henikoff SJNar (2003). SIFT: predicting amino acid changes that affect protein function. Nucleic Acids Research.

[ref-38] Pettersen EF, Goddard TD, Huang CC, Couch GS, Greenblatt DM, Meng EC, Ferrin TE (2004). UCSF Chimera—a visualization system for exploratory research and analysis. Journal of Computational Chemistry.

[ref-39] Quigley HA, Broman AT (2006). The number of people with glaucoma worldwide in 2010 and 2020. British Journal of Ophthalmology.

[ref-40] Rashid M, Yousaf S, Sheikh SA, Sajid Z, Shabbir AS, Kausar T, Tariq N, Usman M, Shaikh RS, Ali M, Bukhari SA, Waryah AM, Qasim M, Riazuddin S, Ahmed ZM (2019). Identities and frequencies of variants in CYP1B1 causing primary congenital glaucoma in Pakistan. Molecular Vision.

[ref-41] Sarfarazi M, Akarsu AN, Hossain A, Turacli ME, Aktan SG, Barsoum-Homsy M, Chevrette L, Sayli BS (1995). Assignment of a locus (GLC3A) for primary congenital glaucoma (Buphthalmos) to 2p21 and evidence for genetic heterogeneity. Genomics.

[ref-42] Shahid M, Azfaralariff A, Law D, Najm AA, Sanusi SA, Lim SJ, Cheah YH, Fazry S (2021). Comprehensive computational target fishing approach to identify Xanthorrhizol putative targets. Scientific Reports.

[ref-43] Shahid M, Azfaralariff A, Zubair M, Abdul kareem Najm A, Khalili N, Law D, Firasat S, Fazry S (2022). In silico study of missense variants of FANCA, FANCC and FANCG genes reveals high risk deleterious alleles predisposing to Fanconi anemia pathogenesis. Gene.

[ref-44] Shahid M, Firasat S, Satti HS, Satti TM, Ghafoor T, Sharif I, Afshan K (2020). Screening of the FANCA gene mutational hotspots in the Pakistani fanconi anemia patients revealed 19 sequence variations. Congenital Anomalies.

[ref-45] Shannon P, Markiel A, Ozier O, Baliga NS, Wang JT, Ramage D, Amin N, Schwikowski B, Ideker T (2003). Cytoscape: a software environment for integrated models of biomolecular interaction networks. Genome Research.

[ref-46] Stoilov I, Akarsu AN, Alozie I, Child A, Barsoum-Homsy M, Turacli ME, Or M, Lewis RA, Ozdemir N, Brice GJTAJohg (1998). Sequence analysis and homology modeling suggest that primary congenital glaucoma on 2p21 results from mutations disrupting either the hinge region or the conserved core structures of cytochrome P4501B1. The American Journal of Human Genetics.

[ref-47] Stoilov I, Akarsu AN, Sarfarazi MJHmg (1997). Identification of three different truncating mutations in cytochrome P4501B1 (CYP1B1) as the principal cause of primary congenital glaucoma (Buphthalmos) in families linked to the GLC3A locus on chromosome 2p21. Human Molecular Genetics.

[ref-48] Stoilov I, Sarfarazi MJIo, science v (2002). The third genetic locus (GLC3C) for primary congenital glaucoma (PCG) maps to chromosome 14q24.3. Investigative Ophthalmology & Visual Science.

[ref-49] Sunkar S, Neeharika DJG (2020). CYP2R1 and CYP27A1 genes: an in silico approach to identify the deleterious mutations, impact on structure and their differential expression in disease conditions. Genomics.

[ref-50] Szklarczyk D, Gable AL, Nastou KC, Lyon D, Kirsch R, Pyysalo S, Doncheva NT, Legeay M, Fang T, Bork PJNar (2021). The STRING database in 2021: customizable protein–protein networks, and functional characterization of user-uploaded gene/measurement sets. Nucleic Acids Research.

[ref-51] Tang H, Thomas PDJB (2016). PANTHER-PSEP: predicting disease-causing genetic variants using position-specific evolutionary preservation. Bioinformatics.

[ref-52] Tang Y, Scheef EA, Wang S, Sorenson CM, Marcus CB, Jefcoate CR, Sheibani NJB (2009). CYP1B1 expression promotes the proangiogenic phenotype of endothelium through decreased intracellular oxidative stress and thrombospondin-2 expression. Blood, The Journal of the American Society of Hematology.

[ref-53] Tang YM, Wo YY, Stewart J, Hawkins AL, Griffin CA, Sutter TR, Greenlee WF (1996). Isolation and characterization of the human cytochrome P450 CYP1B1 gene. The Journal of Biological Chemistry.

[ref-54] Tham Y-C, Li X, Wong TY, Quigley HA, Aung T, Cheng C-Y (2014). Global prevalence of glaucoma and projections of glaucoma burden through 2040: a systematic review and meta-analysis. Ophthalmology.

[ref-55] Vasiliou V, Gonzalez FJ (2008). Role of CYP1B1 in glaucoma. Annual Review of Pharmacology and Toxicology.

[ref-56] Warde-Farley D, Donaldson SL, Comes O, Zuberi K, Badrawi R, Chao P, Franz M, Grouios C, Kazi F, Lopes CT, Maitland A, Mostafavi S, Montojo J, Shao Q, Wright G, Bader GD, Morris Q (2010). The GeneMANIA prediction server: biological network integration for gene prioritization and predicting gene function. Nucleic Acids Research.

[ref-57] Wight TN (2002). Versican: a versatile extracellular matrix proteoglycan in cell biology. Current Opinion in Cell Biology.

[ref-58] Zhang H, Zhu J, Wang C, Sun S, Zheng W-M, Bu D (2017). Improving prediction of burial state of residues by exploiting correlation among residues. BMC Bioinformatics.

[ref-59] Zhang Y (2008). I-TASSER server for protein 3D structure prediction. BMC Bioinformatics.

[ref-60] Zhang Y, Skolnick J (2005). TM-align: a protein structure alignment algorithm based on the TM-score. Nucleic Acids Research.

