# Supplementary figures and images for: Screening of high-risk deleterious missense variations in the CYP1B1 gene implicated in the pathogenesis of primary congenital glaucoma: A comprehensive in silico approach
Source: PeerJ. 2022 Nov 30;10:e14132. doi: 10.7717/peerj.14132 (PMC9744154; doi:10.7717/peerj.14132)

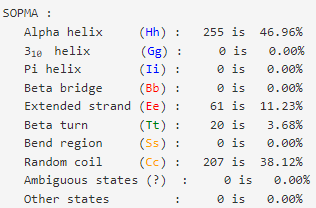

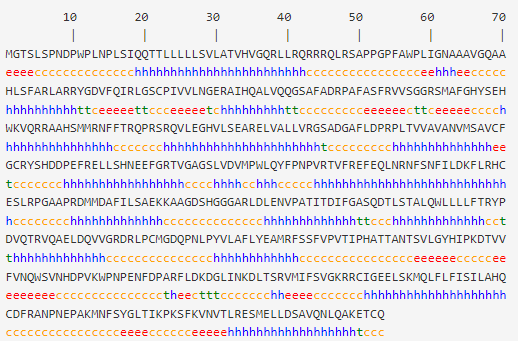
 Supplementary Fig.S1

CYP1B1 SOPMA results

Supplement: Supplemental Information 5 [file peerj-10-14132-s005.docx]
